# Supplementary material for: Complexome profiling of the Chlamydomonas psb28 mutant reveals TEF5 as an early PSII assembly factor
Source: Plant Cell. 2025 Mar 17;37(6):koaf055. doi: 10.1093/plcell/koaf055 (PMC12164590; doi:10.1093/plcell/koaf055)
Supplement: koaf055_Supplementary_Data [file koaf055_supplementary_data.zip › psb28_interactive.zip!/plots/intensity/qualityPlots/peptides/_Cre03.g164700.t1.2.html]

### Cre03.g164700.t1.2

Trivial name:   
SeqCoverage [median]: 20.40  
Mass: 35.58 kDa  
  
 

| Sequence | MissedCleavage | Mass [Da] | wtIntensity | muIntensity | Score |
| --- | --- | --- | --- | --- | --- |
| AAGRLDEYWAAPGR | 1 | 1531.75 | 0.00 | 952.88 | 51.21 |
| ALEQSLGQK | 0 | 972.52 | 472.81 | 1308.97 | 118.33 |
| EATYQR | 0 | 766.36 | 0.00 | 854.35 | 84.61 |
| KFGADASK | 1 | 822.42 | 0.00 | 382.56 | 79.47 |
|  |  |  | 472.8 | 3498.8 |  |
